# Knowledge, Attitudes, and Practices of Cattle Farmers Regarding Antibiotic Use and Antimicrobial Resistance in Selected Districts of Zambia

**DOI:** 10.3390/antibiotics15070651

**Published:** 2026-06-30

**Authors:** Doreen Chilolo Sitali, Geoffrey Mainda, Isaac Silwamba, Inyambo Mumbula, Taona Sinyawa, Fusya Yvonne Goma, Steward Mudenda, Mercy Mukuma, Geoffrey Chomba, Niwael Jesse Mtui Malamsha, Suze Percy Filippini, John Bwalya Muma

**Affiliations:** 1 Department of Health Promotion, School of Public Health, University of Zambia, Lusaka P.O. Box 50110, Zambia; inyambo.mumbula@unza.ac.zm; 2 Food and Agriculture Organization of the United Nations, Lusaka P.O. Box 30563, Zambia; geoffrey.mainda@fao.org (G.M.); geoffrey.chomba@fao.org (G.C.); niwael.mtuimalamsha@fao.org (N.J.M.M.); suze.filippini@fao.org (S.P.F.); 3 Department of Disease Control, School of Veterinary Medicine, University of Zambia, Lusaka P.O. Box 32379, Zambia; isaacs@livestock.co.zm (I.S.); jmuma@unza.zm (J.B.M.); 4 Central Veterinary Research Institute, Department of Veterinary Services, Ministry of Fisheries and Livestock Development, Lusaka P.O. Box 33980, Zambia; taonasinyawa@gmail.com (T.S.); fusya.goma@mfl.gov.zm (F.Y.G.); 5 Department of Pharmacy, School of Health Sciences, University of Zambia, Lusaka P.O. Box 50110, Zambia; steward.mudenda@unza.zm; 6 Department of Food Science, School of Agricultural Sciences and Nutrition, University of Zambia, Lusaka P.O. Box 32379, Zambia; mercy.mukuma@unza.zm

**Keywords:** antimicrobial resistance, antimicrobial use, knowledge, attitudes, practices, small holder livestock systems, Zambia

## Abstract

Background: Antimicrobial resistance (AMR) is increasingly recognized as a major public health challenge in Zambia. However, limited evidence exists on the factors driving AMR and antimicrobial use behaviours among cattle farmers. This study explored farmers’ knowledge, attitudes, and practices (KAPs) regarding AMR and antimicrobial use (AMU), and explored factors influencing them. Methods: Data were collected from Namwala, Mpongwe, and Chingola districts between January and April 2024. A total of 377 cattle farmers participated in a structured survey, supplemented by ten focus group discussions (FGDs) and seventeen in-depth interviews (IDIs). Qualitative data were analysed thematically to identify recurring patterns, while quantitative data were summarized using descriptive statistics and analysed using bivariate tests and regression models to assess key associations. Results: Overall, a small proportion of farmers demonstrated high levels of knowledge (33.9%), positive attitudes (40.4%), and good practices (19.6%) related to AMU and AMR, with significant differences observed across districts. Major drivers of AMU included poor implementation of biosecurity measures, limited access to veterinary services, high reliance on non-prescribed antimicrobials, and weak enforcement of regulations governing antimicrobial distribution. Conclusions: This study highlights critical gaps in AMR-related knowledge and widespread irresponsible AMU among cattle farmers in Zambia. Strengthening targeted AMU/AMR awareness campaigns, improving veterinary service infrastructure, and enhancing regulatory oversight on antibiotic distribution are urgently needed to protect both animal and public health. These findings can support policymakers in designing evidence-based interventions to curb AMR in the livestock sector.

## 1. Introduction

Antimicrobial resistance (AMR) is a growing global health threat, projected to cause an estimated 10 million deaths annually by 2050 if no decisive action is taken [1,2,3]. In the animal sector, antimicrobials play a critical role in preventing, controlling, and treating infectious diseases, thereby enhancing animal productivity and food security. However, widespread misuse and overuse of these drugs have undermined decades of progress in managing infectious diseases in both humans and animals. AMR threatens food production systems, compromises farmers’ livelihoods, and poses risks to food safety. The World Bank further warns that AMR may accelerate global poverty by reducing the Gross Domestic Product (GDP) [4].

In response, the World Health Organization (WHO), the Food and Agriculture Organization (FAO) and the World Organization for Animal Health (OIE) formulated the Global Action Plan (GAP) on AMR, which emphasizes improving public awareness and understanding of AMR. Zambia adopted the UN declaration on AMR and subsequently developed its National Action Plan (NAP) in 2017 [5]. The NAP-AMR focuses on antimicrobial stewardship through strengthened AMR surveillance in human, animal, and environmental sectors; promotion of rational antimicrobial use via standard treatment guidelines and regulatory oversight; and capacity building and awareness to support appropriate prescribing and use of antimicrobials among healthcare and veterinary professionals [5].

Although evidence of AMR in Zambia dates back to the 1960s, its full extent remains unclear [5]. A systematic review by [6] noted that AMR is generally understudied in the country. In addition, Burroughs et al. in [7] reports that unrestricted access to antimicrobials without prescription contributed significantly to AMR in Zambia. This aligns with global evidence that links the misuse and overuse of antimicrobials in humans and animals to the development of AMR [4,8].

Existing studies show that limited knowledge among farmers contributes to inappropriate antimicrobial use [9,10]. Much of the current research focuses on healthcare workers, veterinary staff, drug retailers, and poultry farmers, revealing substantial gaps in knowledge and improper AMU [11,12,13,14]. However, cattle farmers remain underrepresented in AMR research, despite their important role in the livestock sector. In Zambia, the livestock small-holder sector accounts for 42% of the agriculture sector and provides 50% of employment in rural areas [15]. The sector supports rural livelihoods, provides food security, and reduces poverty [16]. Therefore, understanding cattle farmers’ knowledge, practices, and the factors driving antimicrobial use is critical for designing effective public health interventions. This study aimed to:

1. Assess farmers’ knowledge, attitudes, and practices regarding AMR in the Zambian cattle sector.
2. Identify drivers of AMU in beef cattle.
3. Examine infection-prevention practices.

## 2. Results

### 2.1. Quantitative Results

#### 2.1.1. Socio-Demographic and Other Characteristics of Respondents

Three hundred and seventy-seven respondents (377) were interviewed for the survey. Ten focus group discussions (FGDs) and seventeen in-depth interviews (IDIs) were conducted with the selected farmers from three districts, namely Chingola, Namwala, and Mpongwe. Among the respondents, 66 (17%) were from Chingola, 199 (53%) from Namwala, and 112 (30%) from Mpongwe. The median age was 41 years (IQR: 23). The majority were male 340 (90%), married 333 (88%), and full-time farmers 286 (76%). Most had attained secondary education 155 (41%). Nearly half 166 (44%) had over fifteen years of cattle-rearing experience, and 126 (33%) owned more than 20 hectares of land. Significant differences in socio-demographic characteristics were observed across districts, as presented in Table 1.

#### 2.1.2. Knowledge of Antimicrobials and AMR by Respondents

The majority of respondents (59%) had never heard of the term *antimicrobials*. Among those who were familiar with the term, 3% were unable to define what antimicrobials are. Additionally, most respondents who had heard about antimicrobials (59.4%) reported that they had never heard of antibiotic resistance (AMR). However, among those who were aware of antibiotic resistance, all (100%) were able to correctly identify at least one cause and recognized that the misuse of antimicrobials can lead to resistance. Significant differences in knowledge of antimicrobials and AMR were observed across districts, as shown in Table 2.

#### 2.1.3. Attitude of Respondents Towards Antibiotic Use and AMR

The majority of farmers provided positive responses to the attitude-related questions. For instance, 77.4% believed that the misuse of antimicrobials could render them ineffective in treating cattle diseases. Additionally, most respondents (94.1%) considered checking drug expiry dates before purchasing antimicrobials to be important, and 57.7% agreed that AMR can affect both animal and human health. Furthermore, 66.1% of farmers expressed concern about antibiotic resistance (ABR).

Conversely, substantial misconceptions were observed. Most farmers (63.2%) felt that it was acceptable to stop giving antimicrobials if cattle appeared to improve even if the course had not been completed. A large proportion of farmers (79.6%) believed that it was appropriate to use the same antibiotic to treat all types of cattle diseases, and 60.9% felt that it was acceptable to administer antimicrobials to healthy cattle. These findings are summarized in Table 3.

#### 2.1.4. Practices of Respondents Regarding Antibiotic Use and AMR

The majority of respondents reported sharing antimicrobials with other farmers. Most also indicated that they purchased antimicrobials without a prescription (78%). However, 75.1% understood the importance of observing the withdrawal period, and 87.9% stated that they adhered to it. Almost half of the respondents (52.1%) reported consulting veterinary staff when their cattle became ill, while 47.9% did not. Additionally, most farmers (59.5%) indicated that they informed the attending veterinarian when an animal failed to recover after completing a treatment course. Conversely, 11.9% reported that they sold their cattle if there was no improvement following treatment. These findings are presented in Table 4 below.

#### 2.1.5. Farm Management Practices of Respondents

Most respondents reported that they obtained much of their cattle management information from veterinary staff. A large proportion of farmers (95.3%) indicated that they had access to veterinary services, with 47% reporting that these services were provided free of charge. In terms of infection prevention practices, most farms were found to be unfenced. Significant differences among the three districts were observed, as shown in Table 5.

#### 2.1.6. Knowledge, Attitude, and Practice Composite Scores

Overall, only 77 (33.9%) of participants demonstrated a high level of knowledge regarding AMR and AMU. In addition, only 150 (40.4%) participants reported positive attitudes towards antimicrobial resistance and antimicrobial use. Good practices were similarly low, with only 74 (19.6%) of respondents demonstrating appropriate practices in AMU.

Overall, only 33.9% of participants demonstrated a high level of knowledge regarding AMR and AMU. In addition, only 150 respondents (40.4%) reported positive attitudes towards antimicrobial resistance and antimicrobial use. Good practices were similarly low, with only 19.6% of respondents demonstrating appropriate practices in AMU.

#### 2.1.7. Correlations Between KAP Scores

Table 6 presents the Pearson’s correlation results among KAP scores. Overall, weak positive correlations were observed across all domains. Knowledge was weakly correlated with attitudes (r = 0.1255, *p* = 0.0590) and with practices (r = 0.1256, *p* = 0.0594), though these correlations were not statistically significant. In contrast, a statistically significant, albeit weak, positive correlation was found between attitudes and practices (r = 0.1093, *p* = 0.0336).

#### 2.1.8. Infection Prevention Practices of Respondents

##### Most of the Respondents Had Poor Infection Prevention Practices

The majority of respondents (74%) reported that their farms were unfenced, leading to cattle mixing with animals from neighbouring farms (89%). Despite this, most respondents (85%) indicated that they vaccinated their animals at least annually. Significant differences in infection prevention practices were observed across the three districts, with Chingola demonstrating better practices (Table 7).

#### 2.1.9. Common Cattle Diseases and Antimicrobials Used

**Figure 1:**
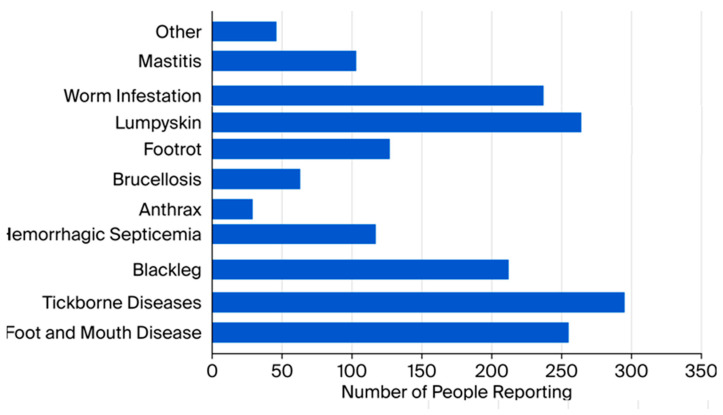
Common diseases affecting cattle in the study areas of Chingola, Mpongwe, and Namwala districts of Zambia.

Most respondents reported that tick-borne diseases were the most common illnesses observed in their cattle (Figure 1), followed by lumpy skin disease and foot-and-mouth disease. These diseases were cited as a major reason for the inappropriate use of antimicrobials by farmers. The antimicrobials most commonly used were penicillin (88.4%) and tetracyclines (11.6%).

#### 2.1.10. Associations of Socio-Demographic Characteristics with Knowledge, Attitudes, and Practices

Table 8 shows the associations between socio-demographic characteristics and respondents’ knowledge, attitudes, and practices. Overall, none of the socio-demographic variables were significantly associated with knowledge or practices (*p* > 0.05). However, experience in rearing cattle was significantly associated with attitudes toward antibiotic use (*p* = 0.006), indicating that respondents with more years of experience were more likely to demonstrate positive attitudes.

#### 2.1.11. Logistic Regression for KAPs

As shown in Table 9, education level and years of experience in cattle rearing were not significantly associated with knowledge (*p* > 0.05). Compared with respondents with no education, those with primary (AOR = 0.844; 95% CI: 0.071–10.098), secondary (AOR = 1.137; 95% CI: 0.096–13.404), and vocational education (AOR = 1.774; 95% CI: 0.147–21.383) did not demonstrate significantly different odds of having appropriate knowledge. Similarly, years of experience in cattle rearing were not significant predictors of knowledge. The model showed adequate fit based on the Hosmer–Lemeshow test (χ^2^ = 2.89, *p* = 0.7173), although the predictive ability was modest (ROC = 0.5968).

#### 2.1.12. Logistic Regression for Predicting Attitude

Table 10 shows the associations between education level and experience and attitude. Education level was not significantly associated with positive attitudes (*p* > 0.05). However, respondents with 11–15 years of experience in cattle rearing were significantly more likely to demonstrate positive attitudes compared with those with less than five years of experience (AOR = 3.833; 95% CI: 1.644–8.935; *p* = 0.002). Although respondents with 6–10 years of experience showed higher odds of positive attitudes (AOR = 2.103), this association was not statistically significant (*p* = 0.080). The model demonstrated good calibration based on the Hosmer–Lemeshow test (χ^2^ = 1.90, *p* = 0.9652) and acceptable discrimination (ROC = 0.6185).

#### 2.1.13. Logistic Regression Analysis for Practices

As shown in Table 11, male respondents had higher odds of reporting appropriate practices compared with females (AOR = 3.183; 95% CI: 0.937–10.814), although this association was not statistically significant (*p* = 0.064). Engagement in other occupations was associated with lower odds of appropriate practices (AOR = 0.549; 95% CI: 0.272–1.110), but this relationship was also not statistically significant (*p* = 0.095). Notably, respondents with 6–10 years of experience in cattle rearing had significantly lower odds of demonstrating appropriate practices compared with those with less than five years of experience (AOR = 0.355; *p* = 0.035). The model demonstrated good fit according to the Hosmer–Lemeshow test (χ^2^ = 1.29, *p* = 0.8635) and moderate discriminatory power (ROC = 0.6428).

### 2.2. Qualitative Results

Four major themes were derived from the qualitative interviews, namely knowledge of antibiotic use and antibiotic resistance, common practices in the use of antimicrobials, drivers of AMU, and cattle management practices, as shown in Table 12 below.

#### 2.2.1. Knowledge, Attitudes, and Practices Regarding AMU and AMR

It is generally recognized that knowledge influences an individual’s attitudes and practices; higher levels of knowledge are typically associated with more positive attitudes and appropriate health behaviours. In this study, most participants were unable to clearly describe what antimicrobials are or had never heard of the term *“antimicrobials.”* This limited understanding was reflected in the views of most respondents. In one of the FGDs, a participant said:

“*Aah, that word (referring to antimicrobials) Madam is where we are behind……. I have never heard about that term*.”

IDI Chingola

Among the participants who had heard about the term ‘antimicrobials,’ they thought that antimicrobials were pain killers.

“*Just like others indicated, it is the same, and antimicrobials are pain killers*.”

FGD, Mpongwe

Still, others described antimicrobials as drugs that caused milk contamination with chemicals, probably referring to instances when antimicrobial residues were detected in the milk of cattle when delivered to milk collection centres. A few farmers also thought that antimicrobials were drugs that killed worms, while others defined antimicrobials as drugs that increased milk production in cows.

However, the few respondents who had a correct understanding of what antimicrobials are were able to describe them accurately. Some of the narratives were as follows:

“*Antibiotic is a medicine that when you give an animal, it kills germs that cause disease, that is what antimicrobials are*”

FGD Mpongwe

In relation to AMR, most respondents indicated that they had not heard of the concept before. However, a few were able to explain that AMR is when medicines stop working.

“*According to my understanding, antibiotic resistance means that antimicrobials have stopped working.*”

Participant, FGD Mpongwe

Regarding participants’ attitudes, most of them demonstrated positive attitudes, as they were able to relate to the fact that their animals were not responding to treatment as they used to in the past. Respondents were able to recognise their own inappropriate practices that could contribute to AMR. Respondents identified practices such as overuse of antimicrobials, sale of counterfeit or substandard drugs by drug stores, use of unprescribed antimicrobials, improper storage and underdosing of antimicrobials as some of the factors that could contribute to antibiotic resistance (ABR). Respondents shared several practices that could potentially contribute to ABR. Common practices included sharing of antimicrobials with fellow farmers, use of unprescribed antimicrobials, improper storage of antimicrobials, failure to complete antimicrobial course, overuse, failure to follow prescribed instructions, and use of incorrect dosages.

#### 2.2.2. Drivers of Antibiotic Use Among Cattle Farmers?

Farmers commonly used antimicrobials to treat diseases in cattle. The most prevalent diseases identified by farmers included tick-borne diseases, foot and mouth disease, theileriosis (corridor disease), black leg, anaplasmosis, tuberculosis, liver flukes, foot rot, brucellosis, mastitis, and lumpy skin. Often, farmers administered antimicrobials whenever they observed signs of poor feeding, fever, weakness, or whenever they suspected that cattle were sick. A farmer stated, and we quote:

“*If l see that my cattle are looking dull, l see that the hide looks muffled, it does not look healthy and is drooling with saliva at the mouth, it means that l have to administer Hitet and oxyject. Sometimes we also add Megapen if the animal is limping and if it has sores on its hide that is why we administer Megapen also*.”

FDG Mpongwe

Some farmers indicated that they administered antimicrobials to calves after vaccinations, and when they heard about an outbreak of disease in neighbouring herds, in order to protect their own cattle. Furthermore, some farmers routinely administered antimicrobials to their animals before sending them to the grazing floodplains, before the commencement of the rainy season and when animals returned from the floodplains under the transhumance grazing system. Sometimes, antimicrobials were administered to cattle at the change of seasons, commonly at the start of the rains and during the dry season, to prevent common diseases that occurred during these seasons.

#### 2.2.3. Common Practices Contributing to Antibiotic Misuse

The literature has shown that inappropriate use of antimicrobials is a major contributor to AMR. Many farmers demonstrated a number of malpractices that contribute to AMR including overuse, unprescribed use, administration of wrong dosages, sharing antimicrobials with fellow farmers, and wrong routes of administration. A farmer explained this regarding overuse:

“*… antimicrobials, we use them heavily, yes and it is also true that as farmers maybe because of lacking knowledge, it is possible that we misuse or we mismanage drugs by administering to our animals*.”

IDI Namwala

Apart from overusing antimicrobials, some of the farmers used unprescribed antimicrobials because they rarely consulted veterinary staff when the cattle fell sick. One respondent said:

“*The issue of medicines is not working is because we do not know what medicine to use for particular diseases. We just mix medicines, whether an animal has diarrhoea or not, we give anyhow*.”

FGD Mpongwe

As a result of using unprescribed antimicrobials, farmers commonly administered the antimicrobials on their own, using wrong dosages and routes of administration:

*“… The reason why these medicines do not work is because we lack knowledge. Because maybe the medicine is supposed to be administered intramuscular, someone administers anyhow just because he has bought the medicine*……….”

FGD Namwala

We also found that farmers administered wrong dosages of antimicrobials, commonly underdosages, because they had no means of estimating the weights of the animals. It was also common for farmers not to complete antimicrobial course once sick cattle appeared to improve, as indicated by one respondent:

“*Yes, when we see that the animal has recovered, we stop administering the antibiotic even if the course has not finished, without lying; this is what we do*.”

IDI, Namwala

Other malpractices included sharing antimicrobials among farmers when they lacked the money to purchase them. Because sometimes antimicrobials had been half used, sharing drugs led to the administration of an incomplete course to cattle.

#### 2.2.4. Challenges Faced by Farmers in Managing Cattle Diseases

Several challenges were identified by farmers, including poor veterinary infrastructure (limited laboratory services with inadequate testing equipment, inadequate laboratories, lack of accommodation for veterinary staff), inadequate veterinary staff, high cost of drugs/vaccines, poor roads and telephone network, inadequate water sources worsened by the drought, poor farmer education on the use of antimicrobials, lack of dip tanks, poor access to veterinary services, inadequate resources to implement infection prevention practices, inadequate nutrition for cattle.

Functional laboratories are essential for effective cattle disease management. It is recommended that cattle diseases are confirmed by laboratory diagnosis before drugs are prescribed in order to reduce on inappropriate use of antimicrobials. However, farmers observed that they lacked laboratory services in their veterinary camps, as illustrated below:

*“………but the greatest problem is that we have no labs where tests can be done. Because cattle require that when they are sick, fast fast it gets tested by drawing blood and give to the vet to find answers. So, where cattle are reared, there should be a lab. But I have never seen a lab in ………. (named district).”*

FGD…

Due to a lack of laboratory facilities, cattle were mostly treated based on clinical symptoms. Clinical diagnosis was in most times remotely made by veterinary camp officers because of vast veterinary camps, shortage of staff, or lack of logistical support for transport. One respondent explained what routinely happened as illustrated below:

“*Every time the vets give advice over the phone, they ask ‘how is the animal looking?’. The reason is that they do not have means of transport to come to our farms, so if they have to come, you need to get into your own pocket*”

FGD ……… (named district)

This study observed that some veterinary camp officers had to oversee more than one camp due to shortage of staff.

In addition to poor public health infrastructure, farmers complained of the high cost of veterinary drugs and high cost of vaccinating cattle:

“*The challenge that we face is that veterinary drugs are very expensive but the cost of cattle keeps on going down*.”

FGD Namwala

Poor road network to most farm sites and lack of registered Agrovet shops increased the cost of drugs and vaccines. For vaccines requiring a cold chain, farmers had challenges storing them.

In addition, most farmers indicated that they had poor infection prevention capacity because most of them practiced traditional farming. Traditional farm systems are characterized by open communal grazing areas and communal water sources, with no feeding supplementation. Therefore, traditional cattle farming makes infection prevention and control difficult. One farmer stated this when he was asked whether his farm was fenced:

“*Our farm is not fenced, our cattle just move around, they mix with other cattle in the flood plains*.”

IDI, Namwala

#### 2.2.5. Associated Infection Prevention Practices in Cattle Management

Biosecurity measures are known to be effective in reducing the need for antimicrobials in cattle management. Therefore, farmers are encouraged to observe infection prevention and control measures to control cattle diseases. However, very few farmers practised infection prevention and control measures. Majority of farmers practiced traditional cattle keeping which is characterised by heavy dependency on communal grazing areas and water sources. In this study, very few farms were paddocked, fenced, or equipped with foot/wheel baths. Most cattle drank water from communal rivers/streams or nearby dams. Although most farmers reported vaccinating their animals at least once a year, few kept records. The only preventive measure that farmers commonly took was annual cattle vaccination against foot and mouth disease (FMD), which is a free vaccine sponsored by the government. Other annual vaccines conducted by farmers were those for haemorrhagic septicaemia and black leg. Several farmers also reported dipping their animals weekly to control ticks and tick-borne diseases. Most respondents also indicated that they never isolated animals bought from other herds, and cattle movement was not controlled.

## 3. Discussion

This study provides critical insights into the knowledge, attitudes, and practices (KAPs) of cattle farmers regarding AMU and AMR in selected districts of Zambia. The findings reveal significant gaps in knowledge and responsible practices, while also highlighting the drivers of AMU and the existing infection prevention practices within the beef cattle sector. These insights are essential for developing targeted antimicrobial stewardship (AMS) programs in the livestock sector, a core component of the One Health approach to mitigating AMR.

### 3.1. Socio-Demographic Characteristics of Study Participants

The majority of participants were males (90%), with a median age of 41 years (IQR: 23). Most of them were married and were full-time farmers. Most of them had attained lower secondary education (41%). Majority of participants were farm owners with over fifteen years of cattle-rearing experience, many owning more than 20 hectares of land. The socio-demographic characteristics are comparable to those found by other studies, which report higher numbers of married males [14,15]. The aforementioned characteristics indicate that farmer-focused education and training are important and should target male farmers, who are the major decision makers in AMU. Extension services, especially in rural areas should be strengthened too.

### 3.2. Knowledge of Antimicrobials and Antimicrobial Resistance

Overall, both quantitative and qualitative findings revealed low levels of knowledge regarding AMU and AMR among cattle farmers. A substantial majority (59%) of farmers had never heard of the term “antimicrobials,” and among those who had, many misconceived antimicrobials as painkillers or agents associated with milk contamination. A study done in Turkey reported inadequate information regarding antimicrobials by livestock farmers who considered them as antipyretic drugs [17]. Low levels of knowledge regarding AMU and AMR among farmers is a significant barrier to responsible AMU and has been documented in other studies conducted in low- and middle-income countries (LMICs), highlighting limited awareness of AMR among various populations, including farmers [8,9,17,18,19]. Furthermore, a meta-analysis conducted in Africa showed a low level of knowledge on antimicrobials and AMR among the public [20]. Equally, previous research in Zambia indicates low AMR knowledge among healthcare providers, veterinary staff, drug retailers, and poultry farmers [12,14,21]. Our findings extend this knowledge gap to cattle farmers, a population rarely studied, despite their critical role in antibiotic use. The low levels of knowledge reported in most African countries underscore the need for antimicrobial stewardship programs to create awareness and educate farmers on antibiotic resistance and the prudent use of antimicrobials. The few farmers who demonstrated good knowledge of antimicrobials and AMR represent an important entry point for peer-led or community-based educational interventions, as recommended in other studies [17,22]. These findings underscore the critical role that livestock farmers play in the development of AMR. It is therefore important for antimicrobial stewardship programs to invest in awareness campaigns targeted at farmers.

On the contrary, a study in Ethiopia reported good knowledge regarding antimicrobials and AMU among livestock farmers [20]. The difference in findings could be attributed to differences in scoring systems and higher educational level among the farmers in Ethiopia. The majority of farmers in our study attained lower secondary education, compared to those in Ethiopia, who had completed senior secondary school.

### 3.3. Attitudes Regarding Antimicrobials and Antimicrobial Resistance

The majority of farmers had poor attitudes towards AMU and AMR. This finding is comparable to a meta-analysis that reported poor attitudes across selected African countries [23]. Poor attitudes among farmers have the potential to undermine antimicrobial stewardship programs by reducing adherence to good practices and thereby promoting misuse of antimicrobials. The poor attitudes observed across most African countries could be attributed to lack of awareness regarding AMR [24]. Most of the farmers felt that it was correct to discontinue administering antimicrobials once sick cattle improved. This, coupled with administering wrong dosages (often underdosing due to lack of animal weight estimation), creates selective pressure, which fosters antibiotic resistance [4,25]. Farmers also felt that it was acceptable to use the same antimicrobial to treat any cattle disease, and that it was prudent to administer antimicrobials to healthy cattle in order to prevent diseases. These findings are consistent with other studies [26,27]. Misuse and administration of sub-therapeutic doses of antimicrobials contributes to AMR [28]. It is therefore important for antimicrobial stewardship programs to strengthen farmer education and focus on behaviour change interventions. Antimicrobial stewardship programs should also advocate for the provision of stronger veterinary oversight.

Contrary to our findings, a meta-analysis that was conducted in Ethiopia among the general public showed favourable attitudes towards AMR and AMU, although the attitudes did not translate into good practices [29]. Qualitative information indicated that farmers’ attitudes were shaped by their long traditional experience in cattle rearing, fostering confidence to administer treatments without consulting veterinary professionals. Farmers also used traditional knowledge to diagnose and sometimes used herbal remedies to treat cattle. Similar findings were observed in a study conducted in India, where farmers used home remedies to treat cattle diseases [30].

### 3.4. Practices of Antibiotic Usage Among Cattle Farmers

This study found that most cattle farmers demonstrated poor practices. The practices included sharing of medications when a fellow farmer did not have money to buy, the use of unprescribed antimicrobials, the administration of wrong dosages (often underdosing due to lack of animal weight estimation), incorrect administration, purchasing of antimicrobials from unregistered Agrovet outlets, and failure to seek veterinary consultations. These findings are consistent with global concerns that the misuse and overuse of antimicrobials, particularly in the animal sector, significantly accelerate AMR [8,9]. Similar trends were reported in other studies, emphasizing the widespread accessibility of veterinary antimicrobials without professional oversight [29,30,31].

Furthermore, most farmers commonly stopped administering antimicrobials once animals appeared to recover. This, coupled with the administration of wrong dosages creates selective pressure, which fosters resistance [32]. The practice of sharing antimicrobials among farmers, often due to financial constraints, further contributes to incomplete courses and inappropriate use, facilitating the spread of resistance. The absence of diagnostic tests before treatment was widespread, leading to empirical treatment based solely on observed symptoms. Lack of diagnostic facilities for testing animal diseases contributes to irrational use of antimicrobials, as found in other African studies [33]. This blind prescribing of antimicrobials, without identifying the causative agent or its susceptibility, is a significant contributor to AMR. It is therefore recommended that antimicrobial stewardship programs invest in veterinary infrastructure and strengthen oversight among cattle farmers. In addition, it is important to strengthen implementation of veterinary regulatory frameworks.

While the survey indicated that a high proportion (87.9%) of farmers observed antimicrobial withdrawal periods, and that 75.1% understood its importance, the qualitative narratives indicated that majority of farmers were aware out withdrawal periods but never observed them. Farmers reported that, when they suspected that their milk had antimicrobial residues, they sold the milk at public markets and in neighbourhoods where milk testing was not done, rather than at the milk collection centres (MCCs). They also reported that they sold animals that did not seem to recover to local butcheries, even before the withdrawal period was over, in order to avoid economic losses. This finding was different from a study conducted in Vietnam among livestock and aquaculture farmers, who understood and observed the withdrawal periods. However, the study in Vietnam used a survey design. Cross-sectional studies are prone to self-reported bias and social desirability, which could lead to falsified higher levels of adherence. The observed contradictions may be attributed to self-reporting and social desirability biases, which frequently affect survey-based studies. Such inconsistencies highlight inherent methodological limitations in relying solely on self-reported data. These findings underscore the need for pragmatic and context-sensitive approaches that build trust between researchers and participants, thereby improving response accuracy. Furthermore, the integration of multiple data collection methods is crucial to enhance the credibility, dependability, and overall rigor of the study findings. The fact that only half (52.1%) of the farmers consulted veterinary staff when animals fell ill, with the other half not doing so, underscores the critical need to improve access to and utilization of professional veterinary services.

### 3.5. Drivers of Antimicrobial Use

Several key drivers of AMU among cattle farmers emerged. Common livestock diseases, particularly tick-borne diseases, were the primary triggers for antibiotic administration. Farmers frequently used antimicrobials whenever they observed non-specific signs of illness such as “poor feeding, fever, weakness, or whenever they suspected that cattle were sick.”. In addition, farmers reported that they administered antimicrobials to calves after vaccinations, during disease outbreaks in neighbouring herds, before sending cattle to grazing floodplains, at the beginning of the rainy season, and upon return from transhumance grazing systems. These practices, while seemingly aimed at prevention, contribute to the overuse of antimicrobials in healthy animals, increasing selective pressure for resistance [4]. The most commonly used antimicrobial was reported to be penicillin (84%), followed by tetracycline (11%). This finding differs from others that found tetracycline to be the most commonly used antibiotic [33,34]. The use of antimicrobials for prophylaxis, often without professional veterinary guidance, has been reported in other studies and is a driver of AMR [20,35]. Therefore, farmer education and training, as well as heightened guidance and supervision by veterinary personnel are key to mitigating poor practices.

### 3.6. Challenges Faced by Farmers in Cattle Management

Limited access to veterinary services and the high cost of drugs/vaccines pushed farmers towards self-medication and reliance on non-prescribed sources. Farmers reported that they “rarely consulted veterinary staff when cattle fell sick”. This was compounded by inadequate enforcement of regulatory controls on antimicrobial distribution, allowing easy access to antimicrobials, without professional oversight from agrovets. A study conducted in Rwanda by [32] also reported limited access to veterinary services among cattle farmers, a factor that could contribute to low KAPs regarding AMU and AMR among the farmers. It is widely recognized that inadequate veterinary services are a major contributor to AMR in resource-limited settings. A study in India reported that structural challenges cause farmers to use alternative solutions that ultimately contribute to development of AMR [36]. It is therefore important that national global action plans at the country level consider infrastructure challenges.

### 3.7. Infection Prevention Practices Among Cattle Farmers

The majority of farmers demonstrated poor infection prevention and control (IPC) practices. The prevalence of traditional, communal cattle keeping, unfenced farms, and cattle mixing with neighbouring herds created ideal conditions for disease transmission, consequently increasing the need for AMU. The lack of foot/wheel baths and the infrequent isolation of newly acquired animals further exacerbate disease spread. Existing studies have demonstrated that improving biosecurity practices are a vital tool in reducing the need for AMU [32,36,37]. While annual vaccination programs (foot and mouth, haemorrhagic septicaemia, black leg) and regular dipping for tick control were reported as common preventive measures, these alone are insufficient to mitigate the high risk of infection in communal grazing systems. The significant differences in infection and prevention and control (IPC) practices across districts, with Chingola demonstrating better practices, likely reflect the demographic variations, with Chingola having more commercial farmers who may have greater resources and awareness regarding farm biosecurity. Zambia is dominated by smallholder livestock farmers who constitute about 80% of the industry. The majority of cattle farmers practice communal, open grazing systems that make implementation of biosecurity measures difficult. Poor IPC directly contributes to increased transmission of infection, accelerated AMR, and reduced effectiveness of antimicrobial stewardship programs.

### 3.8. Study Strengths and Limitations

This study employed a mixed-methods approach, providing rich insights. However, its cross-sectional design limits the ability to establish causal relationships or track changes in KAPs over time. The reliance on self-reported data for practices may be subject to social desirability bias. While selected districts offer diverse farming contexts, the findings may not be fully generalizable to all cattle farming regions in Zambia.

## 4. Materials and Methods

### 4.1. Study Design

A concurrent, convergent mixed-methods design was used, in which quantitative and qualitative data were collected and analysed at the same time. Qualitative and qualitative results were converged during discussion. A pragmatic paradigm was taken in order for the qualitative results to give meaning and context to the quantitative findings.

### 4.2. Study Sites

**Figure 2:**
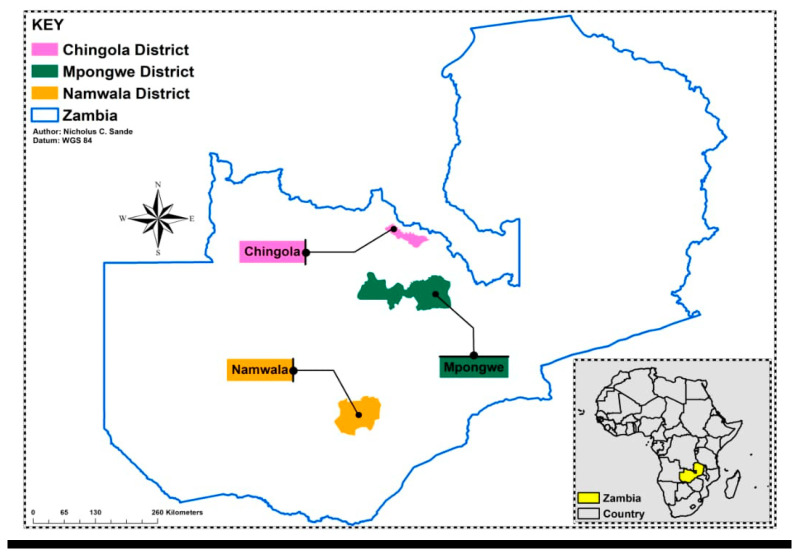
Map showing study sites.

Three sites were conveniently selected namely, Namwala, Mpongwe and Chingola districts of Zambia. The districts were conveniently selected based on recommendation from the Ministry of Fisheries and Livestock, Antimicrobial Stewardship Program. The study period was from between January 2024 to April 2024. The Figure 2 below shows the study areas.

### 4.3. Inclusion/Exclusion Criteria

All cattle farmers that were listed in the antimicrobial stewardship program were included in this study. Records of cattle farmers are routinely maintained by veterinary camp officers (a veterinary camp is a basic unit of veterinary services in a district supervised by a veterinary camp officer). However, farmers who had less than two years’ experience in cattle rearing, those who were below 18 years (age of consent), and those who declined to participate were excluded from the study.

### 4.4. Sampling Strategy

Total enumeration of all eligible farmers registered in the camp registers was done; thus, 377 cattle farmers participated in the survey. For the qualitative arm, purposive sampling was used to identify participants for the qualitative arm from among the survey participants. Therefore, twenty-seven interviews were conducted. Of the twenty-seven interviews, seventeen (17) were in-depth interviews (IDIs) while ten (10) were focus group discussions (FGDs). The interviews were divided as follows: Namwala: three (3) FGDs and ten (10) IDIs; Mpongwe seven (7) FGDs and two IDIs; and Chingola: five (5) IDIs.

### 4.5. Data Collection Procedures

A structured questionnaire was used to collect quantitative data regarding farmers’ levels of knowledge, attitudes, and practices towards ABR and AMU. On the other hand, unstructured interview guides were used to facilitate FGDs and IDIs to explore the drivers of antimicrobial use and the common diseases that drove AMU. The interview guide had eight open-ended questions covering five thematic areas, namely knowledge (two questions), attitudes (one question), practices (two questions), infection prevention (one question), and challenges faced in cattle management (two questions). Farmers were asked to share their knowledge on antimicrobials. To explore attitudes, farmers were asked about their opinions regarding using antimicrobials on their farm and what effects they thought antimicrobials had on humans and cattle. In addition, farmers were asked to share information on how they managed their cattle, who they consulted when their animals were sick, and how they obtained antimicrobials. These questions were meant to assess practices of farmers. Furthermore, questions were asked regarding infection prevention measures that the farmers instituted on their farms and lastly, the challenges they faced in managing their cattle. Face-to-face interviews were used to administer the survey questionnaire and interview guides. IDIs and FGDs were audio-recorded and supplemented with field notes.

#### Data Analysis

Using Dedoose software (version 9.2.22) for Windows, qualitative data was analysed using deductive and inductive thematic analyses. Thematic analysis is the process of identifying patterns or themes within qualitative data. Inductive thematic analysis means that no outside preconceived ideas will influence the data outcomes, and all data deductions come from the data itself [38]. At the end of each field day, recorded audio narratives were reviewed, and adjustments to the questions on the interview guides were made as needed until saturation was achieved. Audio recordings were then transcribed verbatim into Word file documents and later translated into English. The transcribed documents were exported to Dedoose and read and re-read for data internalisation. The responses from each interview resource were coded to generate preliminary codes. Using the preliminary codes, similar codes were clustered into broad categories. The broad categories were then merged into sub-themes, and finally, the themes were analysed to interpret their meaning. Results were then presented with illustrations from relevant verbatim quotes.

STATA version 15 was used to analyse the quantitative data, using descriptive statistics and chi-square analysis to test the association between variables across districts. To determine levels of knowledge, attitudes, and practices, composite scores for each variable were generated. Cut-off points for each of the composite variables (knowledge, attitude, and practices) were determined by computing median scores for each category. Thirteen questions were used to assess respondents’ knowledge. Knowledge levels were categorized as *good* or *poor*. A score of 1 was given for a correct response and zero for a wrong one. A median score of at least 60%, equivalent to eight out of thirteen correct responses, was considered to indicate a high level of knowledge, while scores below 60% were classified as having a low level of knowledge. Attitude was categorised as either positive or negative. A total of twelve questions were asked to assess attitude. One mark was given for each correct response and zero for a wrong one. A median score of 58% (seven out of twelve) was considered positive, while a score below 58% was considered negative. In addition, seventeen questions were used to assess the practices of respondents. A median score of 65%, or a minimum of eleven correct answers, was considered to indicate good practices, while a score below 65% was considered to indicate poor practices. Pearson correlation was conducted to test associations between composite knowledge levels, attitudes, and practices. The level of significance was set at *p* < 0.005. A multivariate logistic regression analysis was conducted to determine the influence of socio-demographic characteristics and other factors on Ks. Initially, univariate analyses were performed to identify variables significantly associated with KAP scores, at a *p*-value ≤ 0.20. Prior to the multivariable analysis, the variance inflation factor (VIF) was calculated to assess multicollinearity among independent variables. The Hosmer–Lemeshow goodness-of-fit test was then applied to evaluate model fit. Finally, the multivariable models were constructed using a forward stepwise selection approach within the generalized linear model (GLM) framework.

## 5. Ethical Approval

This study was developed from a commissioned government antimicrobial stewardship (AMS) program by the Ministry of Fisheries and Livestock through funding from the Food and Agriculture Organization (FAO Project Symbol: OSRO/ZAM/200/USA). The aim of the program was to understand the prevailing practices and drivers of AMR and AMU in the country. Permission to collect data was granted through the office of the Permanent Secretary, Ministry of Fisheries and Livestock to the provincial headquarters and then the study districts. Permissions were also sought from the district coordinators. Respondents were recruited into the program after the purpose of this study was explained, after which consent was obtained. Participation in the program was voluntary. Personal identifiers such as names of farmers, their farms, or residential addresses were not obtained to maintain the confidentiality of information. Respondents for the FGDs and IDIs were assigned pseudonym names or numbers to maintain confidentiality. During the FGDs, respondents were encouraged to avoid sharing information regarding other respondents outside the group. Permission to take photos during data collection was sought from the respondents and faces were blurred to avoid identifying the respondents.

## 6. Conclusions

This study reveals a significant gap in AMR knowledge and responsible AMU among cattle farmers in Zambia, highlighting widespread inappropriate practices driven by common diseases, limited veterinary access, and inadequate regulatory enforcement. The findings provide robust evidence base for policymakers and stakeholders to design and implement urgent, context-specific AMS interventions. These programs must focus on enhancing farmer education, improving access to professional veterinary services and diagnostics, enforcing regulations on antibiotic sales, and promoting robust infection prevention practices to safeguard both animal and public health in Zambia.

## Figures and Tables

**Table 1:** Socio-demographic and other characteristics of respondents.

Variable	District			Total	*p*-Value
	Chingola n (%)	Namwala n (%)	Mpongwe n (%)	n (%)	
**Sex**					
Male Female	52 (78.8)	183 (92.4)	105 (92.9)	340 (90.1)	0.009
	14 (21.2)	15 (7.6)	8 (7.1)	37 (9.8)	
**Total**	66 (100)	198 (100)	113 (100)	377 (100)	
**Marital status**					
Married	51 (77.3)	183 (92)	99 (88.4)	333 (88.3)	0.022
Single	15 (22.7)	16 (8)	13 (11.6)	44 (11.7)	
**Total**	66 (100)	199	112 (100)	377 (100)	
**Educational level**					
No education	1(1.5)	2 (1)	1 (0.8)	4 (1.1)	0.000
Primary	5 (7.5)	84 (42.2)	57 (50.9)	146 38.7)	
Secondary	16 (24.2)	90 (45.2)	49 (43.7)	155 (41.1)	
Tertiary/college	44 (66.7)	23 (11.5)	2 (1.8)	69 (18.3)	
Vocational	0 (0)	0 (0)	3 (2.7)	3 (0.8)	
**Total**	66 (100)	199 (100)	112 (100)	377 (100)	
**Experience in rearing cattle (years)**					
Below 5	5 (7.5)	19 (9.5)	18 (16.2)	42 (11.2)	0.005
6–10	19 (28.3)	42 (21.1)	30 (27)	91 (24.3)	
11–15	23 (34.3)	33 (16.6)	22 (19.8)	76 (20.3)	
Above 15	20 (29.8)	105 (52.8)	41 (36.9)	166 (44.2)	
**Total**	67 (100)	199 (100)	111 (100)	377 (100)	
**Farm size (hectares)**					
Less than 5	12 (18.7)	68 (32.4)	20 (25.2)	100 (26.5)	0.000
6–10	11 (17.2)	46 (21.9)	18 (21.5)	75 (19.9)	
11–15	4 (6.2)	10 (4.8)	23 (6.8)	37 (9.8)	
16–20	7 (10.9)	9 (4.3)	19 (7.7)	35 (9.3)	
Above 20	30 (46.9)	77 (36.6)	23 (38.8)	130 (34.5)	
**Total**	64 (100)	210 (100)	103 (100)	377 (100)	
**Other occupation**					
Yes	22 (33.3)	57 (28.8)	11 (9.8)	91 (24.1)	0.001
No	44 (66.7)	141 (71.2)	101 (90.2)	286 (75.9)	
**Total**	66 (100)	198 (100)	112 (100)	377 (100)	
**Position at farm**					
Farm owner	53 (80.3)	174 (87.9)	82 (73.2)	309 (82)	0.000
Caretaker	2 (3)	17 (8.6)	20 (17.8)	39 (10.3)	
Farm manager	7 (10.6)	4 (2)	9 (8)	20 (5.3)	
Farm worker	4 (6.1)	3 (1.5)	1 (1)	9 (2.4)	
**Total**	66 (100)	198 (100)	112 (100)	377 (100)	

**Table 2:** Knowledge of respondents regarding antimicrobials and AMU.

Variable	District			Total (n)	*p*-Value
	Chingola n (%)	Namwala n (%)	Mpongwe n (%)	Total n (%)	
**Heard about antimicrobials**					
**Yes**	53 (80.3)	118 (59.3)	54 (48.2)	225 (59.7)	0.000
**No**	13 (19.7))	81 (40.7)	58 (51.8)	152 (40.3)	
**Total**	66 (100)	199 (100)	112 (100)	377 (100)	
**What is an antimicrobial?**					
**Wrong response**	0 (0)	0 (0)	5 (9.6)	5 (3)	0.000
**Correct response**	53 (100)	118 (100)	49 (90.7)	220 (97)	
**Total**	53(100)	118 (100)	54 (100)	225 (100)	
**Correctly identify at least one antimicrobial**					
**Yes**	53 (100)	118 (100)	47 (90.4)	218 (96.9)	0.001
**No**	0 (0)	0 (0)	5 (9.6)	5 (2.2)	
**Total**	53 (100)	118 (100)	54 (100)	225 (100)	
**Do antimicrobials kill viruses?**					
**Yes**	25 (47.1)	48 (40.7)	13 (24.0)	87 (40.1)	0.036
**No**	28 (52.8)	70 (59.3)	41 (75.9)	130 (59.9)	
**Total**	53 (100)	118 (100)	54 (100)	225 (100)	
**Should antimicrobials be used to treat all cattle diseases?**					
**Yes**	7 (13.2)	9 (7.6)	7 (13)	23 (10.2)	0.402
**No**	46 (86.8)	109 (92.4)	47 (87.0)	202 (89.8)	
**Total**	53 (100)	118 (100)	54 (100)	225 (100)	
**Heard about antimicrobial resistance**					
**Yes**	25 (47.2)	36 (30.5)	17 (31.5)	79 (35)	0.091
**No**	28 (52.8)	82 (69.5)	37 (68.5)	146 (65)	
**Total**	53 (100)	118 (100)	54 (100)	225 (100)	
**What is antimicrobial resistance?**					
**Correct**	22 (88)	33 (89.2)	16 (94.1)	71 (89.9)	0.798
Wrong	3 (12)	4 (10.8)	1 (5.9)	8 (10.1)	
**Total**	25 (100)	37 (100)	17 (100)	79 (100)	
**Identify at least one cause of antimicrobial resistance**					
**Correct**	25 (100)	37 (100)	17 (100)	79 (100)	0.001
**Wrong**	0 (0)	0 (0)	0 (0)	0 (0)	
**Total**	25 (100)	37 (100)	17 (100)	79 (100)	
Antimicrobials should be prescribed by veterinary staff before treating cattle to avoid AMR					
**Yes**	18 (72.0)	30 (81.1)	17 (100)	65 (82.1)	0.064
**No**	7 (28)	7 (18.9)	0 (00)	14 (17.7)	
**Total**	25 (100)	37 (100)	17 (100)	79 (100)	
**Important to give the correct dosage of antimicrobials to avoid AMR**					
**Yes**	24 (96.0)	37 (100.0)	17 (100)	78 (98.7)	0.335
**No**	1 (4)	0 (0)	0 (0)	1 (1.3)	
**Total**	25 (100)	37 (100)	17 (100)	79 (100)	
**Can the misuse of antimicrobials lead to resistance?**					
**Yes**	25 (100)	34 (91.9)	14 (82.4)	73 (92.4)	0.105
**No**	0 (0.0)	3 (8.11)	3 (17.6)	6 (7.6)	
**Total**	25 (100)	37 (100)	17 (100)	79 (100)	
**What is the importance of observing the withdrawal period**					
**Correct**	38 (57.6)	73 (36.68)	45 (40.2)	221 (75)	
**Wrong**	28 (42.4)	126 (63.32)	67 (59.8)	156 (25)	0.011
**Total**	66 (100)	199 (100)	112 (100)	377 (100)	

**Table 3:** Attitudes of respondents regarding antibiotic use and AMR.

Variable	District			Total (n)	*p*-Value
	Chingola n (%)	Namwala n (%)	Mpongwe n (%)	Total n (%)	
**I feel that it is correct to stop giving a full course of antimicrobials to my cattle if symptoms improve**					
**Agree**	13 (19.7)	87 (43.7)	36 (32.1)	136 (36.1)	0.001
**Disagree**	53 (80.3)	112 (56.3)	76 (67.9)	241 (63.9) *	
**Total**	66 (100)	199 (100)	112 (100)	377 (100)	
**I feel that misuse of antimicrobials makes them fail to treat diseases effectively**					
**Agree**	59 (89.4)	144 (72.4)	90 (80.4)	293 (77.7) *	0.011
**Disagree**	7 (10.6)	55 (27.6)	22 (19.6)	84 (22.3)	
**Total**	66 (100)	199 (100)	111 (100)	377 (100)	
**I feel that buying antimicrobials without prescription is not wrong**					
**Agree**	39 (59.1)	172 (86.4)	84 (75)	295 (78.2)	0.000
**Disagree**	27 (40.9)	27 (13.6)	28 (25)	82 (21.8) *	
**Total**	66 (100)	199 (100)	111 (100)	377 (100)	
**I feel that it is important to check for the expiry date of the drug before treating cattle**					
**Agree**	64 (97)	181 (90.9)	110 (98.2)	355 (94.2) *	0.018
**Disagree**	2 (3)	18 (9.1)	2 (1.8)	22 (5.8)	
**Total**	66 (100)	199 (100)	112 (100)	377 (100)	
**I believe that l can use the same antimicrobial to treat all cattle diseases**					
**Agree**	11 (16.7)	49 (24.6)	16 (14.3)	76 (20.2)	0.068
**Disagree**	55 (83.3)	150 (75.4)	96 (85.7)	301 (79.8) *	
**Total**	66 (100)	199 (100)	112 (100)	377 (100)	
**I believe that antimicrobial resistance has no effect on human and animal health**					
**Agree**	22 (33.3)	80 (40.2)	53 (47.3)	155 (41.1)	0.174
**Disagree**	44 (66.7)	119 (59.8)	59 (52.7)	222 (58.9) *	
**Total**	66 (100)	199 (100)	112 (100)	377 (100)	
**I feel that antimicrobial resistance is of no concern to me as a farmer**					
**Agree**	20 (30.3)	64 (32.2)	40 (35.7)	124 (32.9)	0.722
Disagree	46 (69.7)	135 (67.8)	72 (64.3)	253 (67.1) *	
**Total**	66 (100)	199 (100)	112 (100)	377 (100)	
**I feel that healthy cattle can be given antibiotics to prevent diseases**					
**Agree**	15 (22.7)	90 (45.2)	30 (26.8)	135 (35.8)	0.000
**Disagree**	51 (77.3)	109 (54.8)	82 (73.2)	242 (64.2) *	
**Total**	66 (100)	199 (100)	112 (100)	377 (100)	

* Indicates statistical significance of a variable.

**Table 4:** Practices of respondents regarding AMU.

Variable	District			Total (n)	*p*-Value
	Chingola n (%)	Namwala n (%)	Mpongwe n (%)	Total n (%)	
**Able to buy antimicrobials without a prescription at agrovets**					
**Yes**	39 (59.1)	172 (86.4)	84 (75)	295 (78.3)	0.000
**No**	27 (40.9)	27 (13.6)	28 (25)	82 (21.7) *	
**Total**	66 (100)	199 (100)	112 (100)	377 (100)	
**Do you consult a vet when an animal is sick**					
**Yes**	42 (63.6)	86 (43.2)	74 (66.1)	202 (53.6) *	0.000
**No**	24 (36.4)	113 (56.8)	38 (33.9)	175 (46.4)	
**Total**	66 (100)	199 (100)	112 (100)	377 (100)	
**What do you do when a course of antimicrobials finishes without improvement**					
**Report back to vet**	45 (71.2)	113 (56.8)	68 (60.7)	228 (60.5) *	0.003
**Extend the course of antimicrobial treatment**	4 (6.1)	18 (9)	15 (13.4)	37 (9.8)	
**Use a different antimicrobial**	11 (16.7)	35 (17.6)	15 (13.4)	61 (16.2)	
**Seek advice from a friend**	2 (3)	0 (0)	5 (4.5)	7 (1.9)	
**Sell/eat animal**	2 (3)	33 (16.6)	9 (8)	44 (11.7)	
**Total**	66 (100)	199 (100)	112 (100)	377 (100)	
**Adjusted dose or stopped antimicrobial course**					
**Yes**	29 (43.9)	142 (71.36)	41 (36.6)	212 (56.2)	0.000
**No**	37 (56.1)	57 (28.6)	71 (63.4)	165 (43.8) *	
**Total**	66 (100)	199 (100)	112 (100)	377 (100)	
**Do you share antimicrobials with colleagues**					
**Yes**	38 (57.6)	161 (80.9)	97 (86.6)	296 (78.5)	0.000
**No**	28 (42.4)	38 (19.10)	15 (13.4)	81 (21.5) *	
**Total**	66 (100)	199 (100)	112 (100)	377 (100)	
**Do you observe the withdrawal period**					
**Yes**	59 (89.4)	174 (87.4)	99 (88.4)	332 (88.1) *	0.906
**No**	7 (10.6)	25 (12.6)	13 (11.6)	45 (11.9)	
**Total**	66 (100)	199 (100)	112 (100)	377 (100)	

* Indicates statistical significance of a variable.

**Table 5:** Farm management practices of respondents.

Variable	District			Total (n)	*p*-Value
	Chingola n (%)	Namwala n (%)	Mpongwe n (%)	Total n (%)	
**Source of information regarding cattle management**					
**Social media**	0 (0)	5 (2.5)	3 (2.7)	8 (2.1)	0.293
**Vet staff**	59 (89.4)	172 (86.4)	88 (78.6)	319 (84.6)	
**Fellow farmers**	3 (4.5)	11 (5.5)	13 (11.6)	27 (7.2)	
**Agrovet shops**	4 (6.1)	9 (4.5)	8 (7.1)	21 (5.6)	
**Total**	66 (100)	199 (100)	112 (100)	377 (100)	
**Access to vet services**					
**Yes**	63 (95.4)	193 (97)	96 (85.7)	352 (93.4)	0.000
**No**	3 (4.6)	6 (3)	16 (14.3)	25 (6.6)	
**Total**	66 (100)	199 (100)	112 (100)	377 (100)	
**Vet services free**					
**Yes**	28 (42.4)	102 (51.3)	50 (44.6)	180 (47.7)	0.031
**No**	20 (30.3)	57 (28.6)	22 (19.6)	99 (26.3)	
**Not applicable**	18 (27.3)	40 (20.1)	40 (35.7)	98 (26)	
**Total**	66 (100)	199 (100)	112 (100)	377 (100)	
**Farm fenced**					
**Yes**	16 (24.2)	39 (19.6)	4 (3.6)	59 (15.7)	0.000
**No**	40 (60.6)	134 (67.3)	108 (96.4)	282 (74.8)	
**Partially**	10 (15.2)	26 (13.1)	0 (0.0)	36 (9.5)	
**Total**	66 (100)	199 (100)	112 (100)	377 (100)	
**Cattle feeding system**					
**Free range/communal**	66 (54.5)	199 (89.7)	86 (76.8)	351 (93.1)	0.000
**Zero grazing**	0 (0.0)	0 (0.0)	2 (1.2)	2 (0.5)	
**supplementary**	0 (0.0)	0 (0.0)	24 (21.4)	24 (6.4)	
**Total**	66 (100)	199 (100)	112 (100)	377 (100)	
**Water source**					
**River/dam**	53 (80.3)	158 (79.4)	92 (82.1)	303 (80.4)	0.008
**Borehole**	9 (13.6)	34 (17.1)	7 (6.3)	50 (13.3)	
**Well**	4 (6.1)	7 (3.5)	13 (11.6)	24 (6.4)	
**Total**	66	199 (100)	112 (100)	377 (100)	
**Annual vaccination**					
**Yes**	57 (86.4)	185 (93)	88 (78.6)	330 (97.9)	0.001
**No**	9 (13.6)	14 (7)	24 (21.4)	47 (12.5)	
**Total**	66 (100)	199 (100)	112 (100)	377 (100)	
**Source of information regarding cattle management**					
**Social media**	0 (0)	5 (2.5)	3 (2.7)	8 (2.1)	0.293
**Vet staff**	59 (89.4)	172 (86.4)	88 (78.6)	319 (84.6)	
**Fellow farmers**	3 (4.5)	11 (5.5)	13 (11.6)	27 (7.2)	
**Agrovet shops**	4 (6.1)	9 (4.5)	8 (7.1)	21 (5.6)	
**Total**	66 (100)	199 (100)	112 (100)	377 (100)	
**Access to vet services**					
**Yes**	63 (95.4)	193 (97)	96 (85.7)	352 (93.4)	0.000
**No**	3 (4.6)	6 (3)	16 (14.3)	25 (6.6)	
**Total**	66 (100)	199 (100)	112 (100)	377 (100)	
**Vet services free**					
**Yes**	28 (42.4)	102 (51.3)	50 (44.6)	180 (47.7)	0.031
**No**	20 (30.3)	57 (28.6)	22 (19.6)	99 (26.3)	
**Not applicable**	18 (27.3)	40 (20.1)	40 (35.7)	98 (26)	
**Total**	66 (100)	199 (100)	112 (100)	377 (100)	
**Farm fenced**					
**Yes**	16 (24.2)	39 (19.6)	4 (3.6)	59 (15.7)	0.000
**No**	40 (60.6)	134 (67.3)	108 (96.4)	282 (74.8)	
**Partially**	10 (15.2)	26 (13.1)	0 (0.0)	36 (9.5)	
**Total**	66 (100)	199 (100)	112 (100)	377 (100)	
**Cattle feeding system**					
**Free range/communal**	66 (54.5)	199 (89.7)	86 (76.8)	351 (93.1)	0.000
**Zero grazing**	0 (0.0)	0 (0.0)	2 (1.2)	2 (0.5)	
**Supplementary**	0 (0.0)	0 (0.0)	24 (21.4)	24 (6.4)	
**Total**	66 (100)	199 (100)	112 (100)	377 (100)	
**Water source**					
**River/dam**	53 (80.3)	158 (79.4)	92 (82.1)	303 (80.4)	0.008
**Borehole**	9 (13.6)	34 (17.1)	7 (6.3)	50 (13.3)	
**Well**	4 (6.1)	7 (3.5)	13 (11.6)	24 (6.4)	
**Total**	66	199 (100)	112 (100)	377 (100)	
**Annual vaccination**					
**Yes**	57 (86.4)	185 (93)	88 (78.6)	330 (97.9)	0.001
**No**	9 (13.6)	14 (7)	24 (21.4)	47 (12.5)	
**Total**	66 (100)	199 (100)	112 (100)	377 (100)	

**Table 6:** Pearson’s correlation between KAP scores.

Variable	Knowledge	Attitude	Practice
Knowledge			
Pearson’s correlation	1	0.1255	0.1256
Sig. (2-tailed)		0.0590	0.0594
Attitude			
Pearson’s correlation	0.1255	1	0.1093
Sig. (2-tailed)	0.0590		0.0336 *
Practice			
Pearson’s correlation	0.1256	0.1093	1
Sig. (2-tailed)	0.0594	0.0336 *	

* Correlation is significant at the 0.05 level.

**Table 7:** Infection prevention practices of respondents.

Variable	District			Total (n)	*p*-Value
	Chingola n (%)	Namwala n (%)	Mpongwe n (%)	Total n (%)	
**Farm fenced?**					
Yes	49 (73.1)	39 (19.7)	4 (3.6)	92 (24.4)	0.000
No	18 (26.9)	159 (80.3)	108 (96.4)	285 (75.5)	
**Total**	67 (100)	198 (100)	112 (100)	377 (100)	
**Cattle mixes with others**					
Yes	44 (65.7)	188 (94.9)	110 (98.2)	342 (90.7)	0.000
No	23 (34.3)	10 (5)	2 (1.8)	35 (9.3)	
**Total**	67 (100)	198 (100)	112 (100)	377 (100)	
**Annual vaccination program?**					
Yes	57 (85.1)	184 (92.5)	86 (77.5)	327 (86.7)	0.001
No	10 (14.9)	15 (7.5)	25 (22.5)	50 (13.3)	
**Total**	67 (100)	199 (100)	111 (100)	377 (100)	

**Table 8:** Associations of socio-demographic characteristics with knowledge, attitudes, and practices.

Variable	Knowledge (n = 225)		Attitude (n = 377)		Practice (n = 377)	
	Appropriate f (%)	*p*-Value	Positive f (%)	*p*-Value	Appropriate f (%)	*p*-Value
**Sex**						
**Male**	5	0.188	138 (90.8)	0.736	3 (4.1)	0.061
**Female**	72		14 (9.2)		71 (95.9)	
**Marital status**						
**Married**	69 (89.6)	0.936	135 (88.8)	0.982	67 (90.5)	0.602
**Single**	8 (10.4)		17 (11.2)		7 (9.5)	
**Education level**						
**No education**	1 (1.3)	0.413	1 (0.6)	0.256	1 (1.3)	0.964
**Primary**	20 (26)		146 (38.7)		30 (40.5)	
**Secondary**	31 (40.3)		155 (41.1)		29 (39.2)	
**Tertiary/College**	25 (32.4)		3 (0.8)		1 (1.3)	
**Vocational**	0 (0.0)		69 (18.3)		13 (17.6)	
**Farm size (hectares)**						
**Less than 5**	18 (24.7)	0.987	31 (23)	0.462	82 (25.3)	0.884
**6–10**	14 (19.2)		33 (24.4)		70 (21.6)	
**11–15**	6 (8.2)		7 (5.2)		22 (6.8)	
**16–20**	6 (8.2)		8 (5.9)		25 (7.7)	
**Above 20**	29 (39.73)		56 (41.5)		125 (38.6)	
**Experience in rearing cattle (years)**						
**Below 5**	5 (6.6)	0.652	10 (6.6)	0.006 *	11 (14.9)	0.082
**6–10**	15 (19.7)		38 (25)		10 (13.5)	
**11–15**	19 (25)		42 (27.6)		19 (25.7)	
**Above 15**	37 (48.7)		62 (40.8)		34 (45.9)	
**Position on farm**						
**Farm owner**	68 (88.3)	0.557	124 (81.6)	0.931	60 (81)	0.934
**Caretaker**	5 (6.5)		15 (9.9)		7 (9.5)	
**Farm manager**	3 (3.9)		8 (5.3)		5 (6.8)	
**Farm worker**	1 (1.3)		5 (3.3)		2 (2.7)	
**District**						
**Chingola**	22 (28.6)	0.533	31 (20.4)	0.341	13 (17.6)	0.813
**Namwala**	38 (49.3)		74 (48.7)		37 (50.0)	
**Mpongwe**	17 (22.1)		47 (30.9)		24 (32.4)	
**Other occupation**						
**Yes**	24 (31.2)	0.309	32 (21.1)	0.553	11 (15.1)	0.084
**No**	53 (68.83)		120 (78.9)		62 (84.9)	

* Significant association.

**Table 9:** Logistic regression analysis for factors predicting knowledge.

Predictor Variables	Knowledge		
	AOR	*p*-Value	Confidence Interval
Education level			
No education	1		
Primary	0.844	0.894	0.071–10.098
Secondary	1.137	0.919	0.096–13.404
Vocational	1.774	0.652	0.147–21.383
Experience in rearing cattle (years)			
Below 5	1		
6–10	0.6001	0.431	0.169–2.137
11–15	1.047	0.942	0.299–3.662
Above 15	1.083	0.895	0.331–3.545

Hosmer–Lemeshow goodness-of-fit test statistic for knowledge: 2.89, *p* = 0.7173; ROC curve—0.5968.

**Table 10:** Logistic regression analysis for factors predicting attitude.

Predictor Variables	Attitude		
	AOR	*p*-Value	Confidence Interval
Education level			
No education	1(ref)		
Primary	1.729	0.647	0.166–17.965
Secondary	1.841	0.609	0.177–19.086
Tertiary	1.205	0.913	0.042–34.660
Vocational	3.088	0.351	0.289–32.978
Experience in rearing cattle (years)			
Below 5	1 (ref)		
6–10	2.103	0.080	0.915–4.833
11–15	3.833	0.002	1.644–8.935
Above 15	1.835	0.128	0.839–4.011

Hosmer–Lemeshow goodness-of-fit test statistic for knowledge: 1.90, *p* = 0.9652; ROC curve—0.6185.

**Table 11:** Logistic regression analysis for factors predicting practice.

Predictor Variables	Practice		
	AOR	*p*-Value	Confidence Interval
Sex			
Female	1 (ref)		
Male	3.183	0.064	0.937–10.814
Other occupation			
No	1 (ref)		
Yes	0.549	0.095	0.272–1.110
Experience in rearing cattle (years)			
Below 5	1 (ref)		
6–10	0.355	0.035	0.169–2.137
11–15	0.994	0.990	0.299–3.662
Above 15	0.680	0.343	0.331–3.545

Hosmer–Lemeshow goodness-of-fit test statistic for knowledge: 1.29, *p* = 0.8635; ROC curve—0.6428.

**Table 12:** Themes and sub-themes.

Theme	Subtheme	Category
Knowledge of antibiotic use and antibiotic resistance	Knowledge about antimicrobials Knowledge about antibiotic resistance	Knowledge
Common practices in the use of antimicrobials	Misuse of antimicrobials Overuse of antimicrobials Underdosing Incomplete course of antimicrobials Sharing antimicrobials Observance of withdrawal period	Practices in use of antimicrobials
Drivers of antibiotic use	Cattle diseases	Drivers
Cattle management practices	Transhumance grazing River/dam and borehole sources Dipping Vaccination Cattle movement	Feeding methods Water sources Infection prevention practices Disease management

## Data Availability

The data presented in this study are only available on request from the corresponding author. Data can only be made available with permission from the Ministry of Fisheries and Livestock of the government Republic of Zambia.

## Abbreviations

AMR	Antimicrobial resistance
AMU	Antimicrobial use
FAO	Food and Agriculture Organization
FGD	Focus Group Discussion
GAP	Global Action Plan
IDI	In-depth interviews
KAPs	Knowledge, attitude, and practices
MoH	Ministry of Health
WHO	World Health Organization
